# Oxycodone/naloxone versus tapentadol in real-world chronic non-cancer pain management: an observational and pharmacogenetic study

**DOI:** 10.1038/s41598-022-13085-5

**Published:** 2022-06-16

**Authors:** Jordi Barrachina, Cesar Margarit, Javier Muriel, Santiago López-Gil, Vicente López-Gil, Amaya Vara-González, Beatriz Planelles, María-del-Mar Inda, Domingo Morales, Ana M. Peiró

**Affiliations:** 1 Neuropharmacology on Pain (NED), Alicante Institute for Health and Biomedical Research (ISABIAL-FISABIO Foundation), Alicante, Spain; 2 Pain Unit, Department of Health of Alicante - General Hospital, Alicante, Spain; 3 Occupational Observatory, Miguel Hernández University of Elche, Alicante, Spain; 4 Department of Pharmacology, Paediatrics and Organic Chemistry, Miguel Hernández University of Elche, Elche, Spain; 5 Operations Research Centre, Miguel Hernández University of Elche, Elche, Spain; 6 Clinical Pharmacology Unit, Department of Health of Alicante - General Hospital, Alicante, Spain; 7 Neuropharmacology on Pain (NED) Research Group, Hospital General Universitario de Alicante, C/Pintor Baeza, 12, 03010 Alicante, Spain

**Keywords:** Genetics, Neuroscience, Medical research, Biomarkers, Predictive markers, Prognostic markers

## Abstract

Tapentadol (TAP) and oxycodone/naloxone (OXN) potentially offer an improved opioid tolerability. However, real-world studies in chronic non-cancer pain (CNCP) remain scarce. Our aim was to compare effectiveness and security in daily pain practice, together with the influence of pharmacogenetic markers. An observational study was developed with ambulatory test cases under TAP (n = 194) or OXN (n = 175) prescription with controls (prescribed with other opioids (control), n = 216) CNCP patients. Pain intensity and relief, quality of life, morphine equivalent daily doses (MEDD), concomitant analgesic drugs, adverse events (AEs), hospital frequentation and genetic variants of *OPRM1* (rs1799971, A118G) and *COMT* (rs4680, G472A) genes, were analysed. Test CNCP cases evidenced a significantly higher pain relief predictable due to pain intensity and quality of life (R^2^ = 0.3), in front of controls. Here, OXN achieved the greatest pain relief under a 28% higher MEDD, 8–13% higher use of pregabalin and duloxetine, and 23% more prescription change due to pain, compared to TAP. Whilst, TAP yielded a better tolerability due the lower number of 4 [0–6] AEs/patient, in front of OXN. Furthermore, OXN *COMT*-AA homozygotes evidenced higher rates of erythema and vomiting, especially in females. CNCP real-world patients achieved higher pain relief than other traditional opioids with a better tolerability for TAP. Further research is necessary to clarify the potential influence of *COMT* and sex on OXN side-effects.

## Introduction

In last decades, prescription opioids began to be used for chronic non-cancer pain (CNCP) outside the context of palliative medicine^1,2^. Since then, there has been an increase in opioid long-term use for conditions that are beyond the evidence base^3,4^. Efforts are necessary to optimize the opioid benefit/risk balance of opioid use developing best clinical practice guidelines based on researched circumstances^5^.

New, recently marketed opioid with potentially improved tolerability have opened a more optimistic door. Most clinically relevant opioid analgesics bind to μ opioid receptors in the central and peripheral nervous system in an agonist manner to elicit analgesia^6^. However, naloxone, a μ receptor antagonist, combined with oxycodone (OXN), a μ and k-opioid receptor agonist, may improve pain control, minimizing opioid-related bowel dysfunction adverse events (AEs)^7,8^. Another new opioid is tapentadol (TAP), that has a dual mode of action. TAP as a Tapentadol displays μ-opioid receptor agonist and noradrenaline reuptake inhibitor properties and is purported to have comparable analgesic efficacy to controlled-release oxycodone with a better opioid tolerability profile^9^ and fewer pharmacological interactions^10^ due others opioids.

There are several well-designed studies comparing both OXN’s and TAP’s analgesic effects, however some of the results seem contradictory^11,12^. Randomized placebo-controlled studies demonstrated similar analgesic levels between the two in osteoarthritis knee pain cases^13^, and similar tolerability profiles following minor orthopaedic/trauma surgeries^14,15^. However, in other CNCP studies, TAP showed better gastrointestinal tolerance and quality of life improvement than OXN did^16^. The main question is whether this better profile is maintained or changed in real-world pain practice with polymedicated and elderly regular pain patients.

Given this scenario, precision medicine could provide data to understand such variability. *Mu opioid receptors (OPRM)* and *Catechol-O-Methyltransferase (COMT)* are candidate genes with a significant influence on morphine analgesic response^17,18^. There are few validated studies that ultimately call for pharmacogenetic testing to be conducted when initiating opioid therapy in pain management^19–23^. Results have indicated that many favourable analgesic effects may depend on increased OPRM density^24,25^ and on higher dopamine concentrations in the prefrontal cortex^17^. Moreover, the *OPRM*gene has been widely studied for distinct pain sensitivity phenotypes^26^ and it seems that *OPRM* A118G (rs1799971) and *COMT* G1947A (rs4680) heterozygous patients, need significantly less morphine as compared to A118 mutant homozygous ones^27^. What´s more, sex-specific effects that have been detected^9^ could be responsible for modulating the *COMT* genotype’s effects on synaptic dopaminergic concentrations and emotion modulation capabilities^28^. These results imply a complex nature in the genotype-phenotypes’ interactions.

The purpose of present study was to compare OXN and TAP analgesic effects and tolerability in real-world CNCP management. Here, genetic variants’ (*OPRM1* and *COMT* genes) impact on clinical outcomes were analysed.

## Results

Chronic pain was mostly due to lumbago (nonspecific, associated with radiculopathy, spinal stenosis, or another specific spinal cause), followed by knee pain. Nearly half of them had mixed neuropathic-nociceptive symptoms.

### Demographic and clinical outcomes

A summary of the characteristics of the subjects included in the study is presented in Table 1.

**Table 1:** Demographic, clinical, and pharmacological data in chronic non-cancer pain patient’s total population, control, and tapentadol (TAP) and oxycodone/naloxone (OXN) cases groups.

	Total (n = 584)	Control (n = 216)	CASE		p-value Effect size
			TAP (n = 194)	OXN (n = 175)	
Sex (female) (%)	71	69	74	65	0.193
					0.002^I^
Age	65 ± 14	65 ± 13	65 ± 14	64 ± 13	0.845
					0.004^II^
VAS pain intensity (0–100 mm)	64 ± 26	60 ± 27	61 ± 26	64 ± 26	0.402
					0.002^III^
**Likert pain intensity (%)**					
None	4	7	6**	3**	
Mild	10	6	15**	13**	< 0.001
Moderate	29	26	24**	29**	**0.98**^**I**^
Severe	42	34	42**	48**	
Extremely severe	15	27	10**	17**	
VAS pain relief (0–100 mm)	37 ± 29	31 ± 30	36 ± 28**	40 ± 30**	0.006
					0.016^III^
**Likert pain relief (%)**					
None	21				0.007
Mild	28	19	24	32**	0.42^I^
Moderate	37	34	36	35**	
Severe	11	11	11	9**	
Extremely severe	3	6	4	3**	
VAS EuroQol (0–100 mm)	45 ± 22	45 ± 24	45 ± 22	46 ± 23	0.968
					0.004^III^
**Utilization of hospital services (%)**					
Due to pain					
Prescription change	24	11	19	42**^†^	< 0.001
					**0.676**^**I**^
Emergency department visit	21	17	16	22	0.2615
					**1.07**^**I**^
Hospital admission	6	5	4	8	0.385
					0.47^I^
Due to other causes					
Prescription change	21	11	18	26*	< 0.001
					**1.18**^**I**^
Emergency department visit	24	14	27**	25**	< 0.001
					**1.34**^**I**^
Hospital admission	17	7	10	22**	< 0.001
					**1.93**^**I**^

Data is presented as mean ± SD or as %.

Comparison cases vs. control, *p < 0.05, **p < 0.001 and ^†^p < 0.05 tapentadol vs. oxycodone/naloxone, cell in italics. Chi-square χ^2^ the effect size was determined using ^I^Cramer’s V (effect size < 0.2 small, 0.2 < effect size < 0.6 intermediate and effect size > 0.6 large effect).

^II^Eta squared for One-Way ANOVA.

^III^Eta squared for Kruskal Wallis Test (effect size of 0.01–0.04 small, 0.06–0.11 intermediate and 0.14–0.2 large effect).

Large effect size is written in bold font.

Most of CNCP patients were elderly females (65 ± 14 years, 71% women) who presented moderate chronic pain (VAS, 64 ± 26 mm), mild relief (37 ± 29 mm) and moderate QoL (45 ± 22 mm) at the time of inclusion. A total of 3–6% of them had no pain, and 57% had severe or extremely severe pain. Controls suffered around 10% more extreme severe pain and a statistically significant lower pain relief level (VAS, 31 ± 30 mm) as compared to OXN (40 ± 30 mm) and TAP (36 ± 28 mm) test cases (p < 0.001) without any significant differences on pain relief between test cases groups. On the other hand, any significant clinical difference was found between naïve or opioid switchers (Supplementary Table S1).

A significant positive correlation was evidenced between pain relief and QoL in cases with a negative correlation to pain intensity in all groups. Thus, pain intensity and QoL were predictive values of relief (R^2^ = 0.3). Pain relief was negatively affected by age in control´s relief and positively by anxiolytics in OXN group, while sex, number of AEs, MEDD, neuromodulators, and analgesics showed no impact (Supplementary Table S2).

### Utilization of hospital services

Significantly, OXN cases needed double the percentage of prescription changes due to pain as compared to TAP (42% vs. 19%, p = 0.002), as can be seen in Table 1. Due to causes other than pain, OXN cases needed a significant 15% more prescription changes (p < 0.001, large effect size = 1.93, 95% CI [1.2–2.5]), 11% more EDs visits (p < 0.001, large effect size = 1.34. 95% CI [0.7–2.2]) and 15% more hospital admissions than controls (p < 0.001, large effect size = 1.18, 95% CI [0.7–2.02]). Additionally, TAP needed 13% more EDs than controls (p < 0.001, large effect size = 1.93, 95% CI [1.2–2.5]). Globally, cases showed 11–13% more ED visits (p < 0.001, large effect size = 1.34, 95% CI [0.7–2.2]) than controls.

### Pharmacology variables

**Figure 1:**
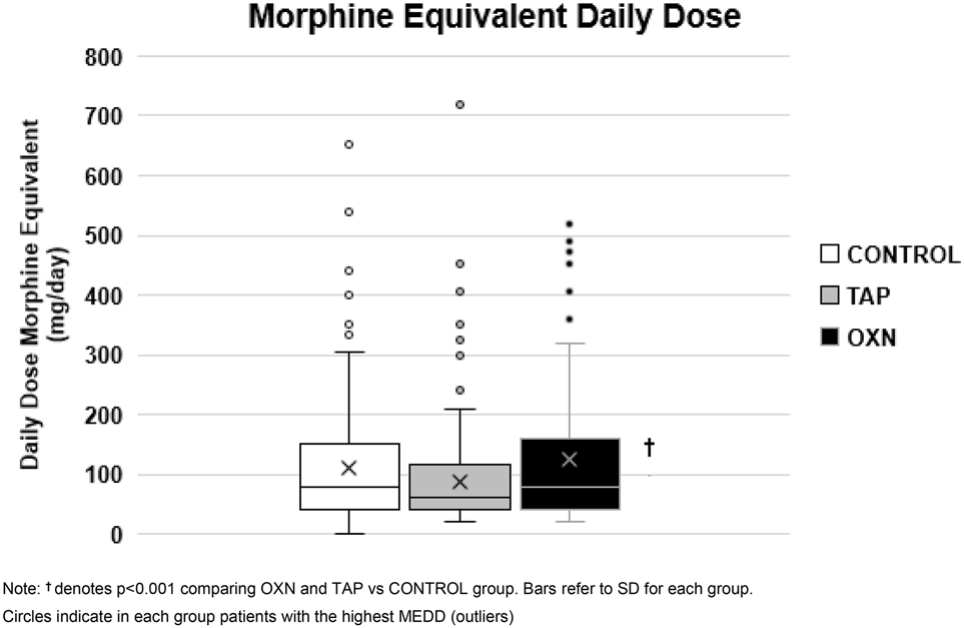
Daily morphine equivalent dose (MEDD) (mean ± SD) depending on control and tapentadol (TAP) and oxycodone/naloxone (OXN) cases groups.

A summary of the prescribed analgesic drugs is presented in Table 2 and Fig. 1.

**Table 2:** Analgesic drug prescription in control, and tapentadol (TAP) and oxycodone/naloxone (OXN) cases groups for chronic non-cancer pain.

Pain medication n (%)	Control (n = 216)	Case		p-value Cramer’s V
		TAP (n = 194)	OXN (n = 175)	
Analgesic	71 (33)	73 (36)	55 (31)	0.329
				0.044^I^
Tramadol	92 (43)	26 (12)*	13 (7)*	< 0.001
				0.381^I^
NSAIDs	23 (11)	25 (12)	22 (12)	0.639
				0.023 ^I^
**Opioids n (%)**				
MEDD (mg/day)	110 ± 109	89 ± 88*	***124* ± *109***^**††**^	0.007
				0.017^II^
Fentanyl transdermal	75 (35)	15 (7)**	***29 (16)***^†^*	< 0.001
				0.194^I^
Oxycodone	28 (13)	6 (4)*	3 (2)*	< 0.001
				0.213^I^
Morphine	27 (12)	12 (6)*	4 (2)*	< 0.001
				0.165^I^
Buprenorphine	23 (11)	3 (2)*	4 (2)*	< 0.001
				0.293^I^
Hydromorphone	14 (6)	2 (1)**	2 (1)**	< 0.001
				0.153^I^
**Coadyuvants n (%)**				
Pregabalin	107 (49)	51 (25)*	***72 (40)***^**†**^	< 0.001
				0.212^I^
Gabapentin	48 (22)	22 (11)*	24 (13)*	0.003
				0.138^I^
Duloxetine	71 (33)	32 (16)**	***44 (24)***^**†**^	< 0.001
				0.167^I^
Benzodiazepines	83 (38)	88 (43)	83 (46)	0.292
				0.064^I^

*MEDD* morphine equivalent daily dose.

Comparison cases vs. control, *p < 0.05, **p < 0.001 and ^†^p < 0.05 tapentadol vs. oxycodone/naloxone, cell in italics denotes also significant differences between tapentadol and oxycodone/naloxone. Effect size was determined as follows: For Chi-square χ^2^ test using ^I^Cramer’s V (effect size < 0.2 small, 0.2 < effect size < 0.6 intermediate and effect size > 0.6 large effect).

^II^Eta squared for One-Way ANOVA (effect size of 0.01–0.04 small, 0.06–0.11 intermediate and 0.14–0.2 large effect).

Large effect size is written in bold font.

OXN showed the highest MEDD requirements (124 ± 109 mg/day), which were 28% higher than TAP’s (89 ± 88 mg/day, p < 0.001) and 11% higher than controls (110 ± 109 mg/day, p > 0.05). Here, TAP showed the lowest MEDD, even significantly lower than controls. In contrast, the controls required higher tramadol use than cases.

**Figure 2:**
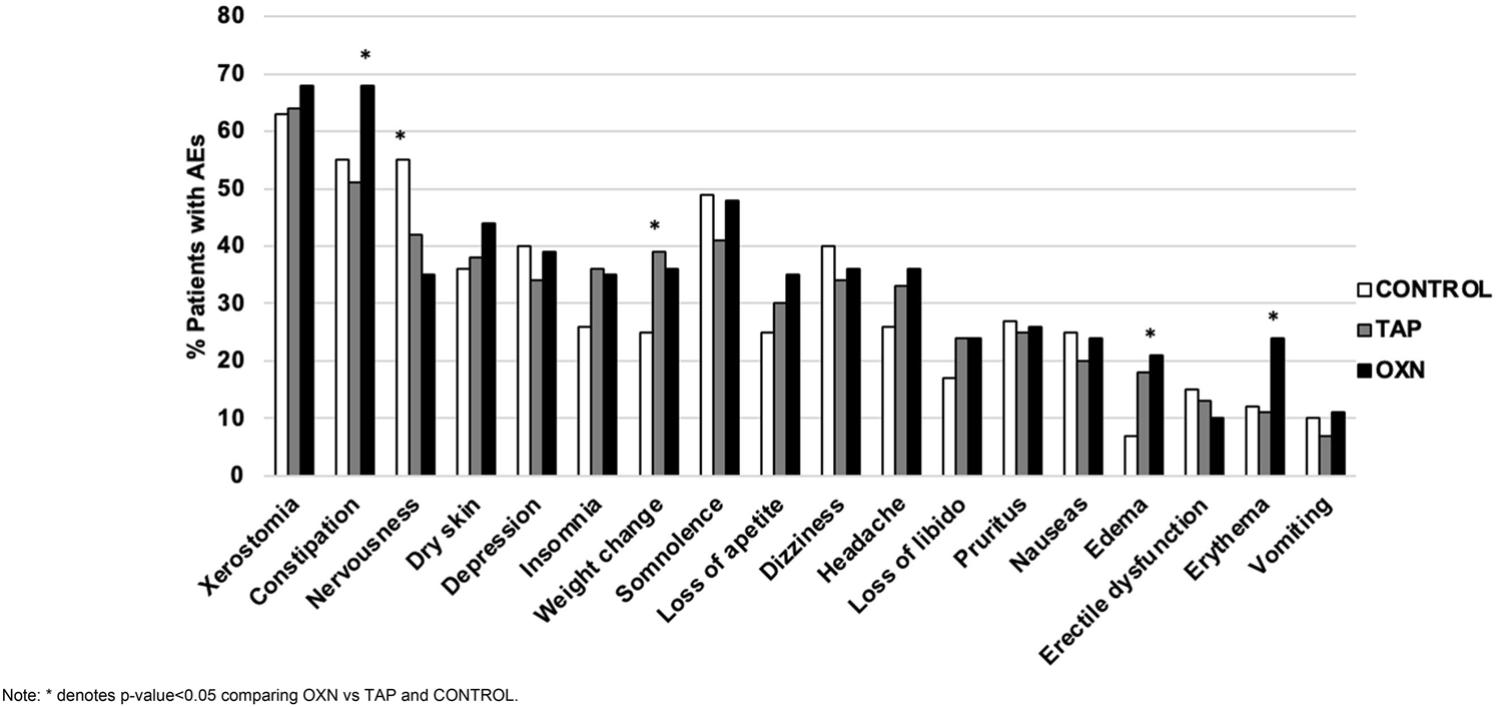
Percentage of patients with adverse events of patients (AEs) self-reported in in total population, control, tapentadol (TAP) and oxycodone/naloxone (OXN) cases groups.

On the other hand, the rates of coadjuvant medication use differed between groups. OXN patients reported a significant 15% higher use of pregabalin (p = 0.002) and 8% higher duloxetine use (p = 0.040), than TAP, as seen at Table 2 and Fig. 2. However, control group needed a significant higher use of 9–24% pregabalin, 9–11% gabapentin and 7–17% of duloxetine (p < 0.001) vs. test cases, but at similar doses requirements. Gabapentin (cases vs. controls, 1600–1800 mg/day vs 1650 [675–2250] mg/day, p = 0.551), antidepressants (above all duloxetine 45 [30–60] mg/day for all groups, p = 0.925), and benzodiazepines (above all lorazepam 3–4 [2–6] mg/day for all groups, p = 0.471) were prescribed in a similar dose-range except for the non-significant higher median dose of pregabalin (cases vs. controls, 300 mg/day vs. 150 [150–300] mg/day, p = 0.236). Other antidepressants (such as amitriptyline or fluoxetine) did not exceed 1–2% of drug prescriptions. Hence, they were not included in the final analysis due to their low prescription rate.

### Safety profile

**Figure 3:**
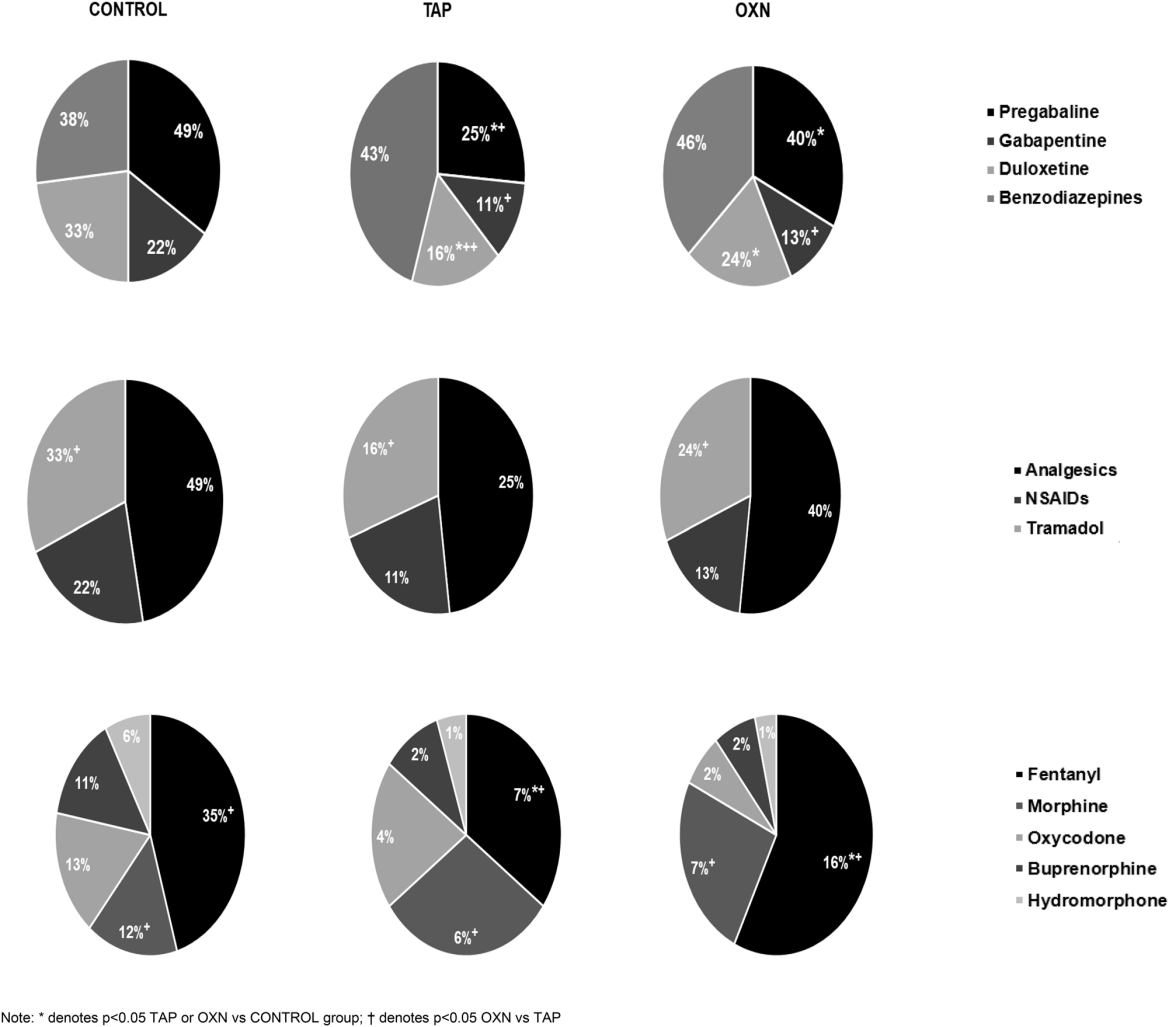
Analgesic, opioid and coadjuvant treatment in total population, control, and case (tapentadol and oxycodone/naloxone) groups.

The percentage of patients showing some AE is displayed in Fig. 3 and in Supplementary Table S3.

In total, 3131 AEs (incidence rate of 5 AEs/patient) were logged through questionnaires, with most being mild and having disappeared with the withdrawal of the drug. Nearly all patients (94%) reported at least one AE with a median of 6 AEs (IQR 3.5–9) per patient. Most prevalent were dry mouth (65%), nervousness (54%) and constipation (46%). According to MEDRA, the most frequents disorders were: 22% (n = 657) psychiatric (40% nervousness, 29% insomnia, 31% depression), 21% (n = 611) nervous (38% somnolence, 30% headache, 32% dizziness) and 16% gastrointestinal (62.6% constipation, 27% nausea, 10% vomiting).

TAP group referred the lowest number of AEs (4 [0–6] AEs/patient) compared with OXN and control groups. In this context, OXN patients reported more 13–17% frequent constipation (p-value ≤ 0.001, large effect size = 2.4, Odd ratio of OXN vs control 2.46 [1.6–3.8] and OXN vs TAP 2.3 [1.5–3.5]), as well as 12% and 11% higher incidence of erythema than both TAP and control patients (p = 0.002, large effect size = 1.46, Odd ratio OXN vs control 2.37 [1.3–4.2] OXN vs TAP 2.47 [1.4–4.3]).

What´s more, controls showed a significant 7% and 20% higher frequency of nervousness vs. TAP and OXN, respectively (p = 0.002, large effect size = 1.461). Control also developed a higher frequency of edema at 11% and 14% compared to TAP and OXN, respectively (p = 0.001, large effect size = 1.498, Odd ratio of TAP vs control 2.65 [1.3–5.2]; OXN vs control 3.4 [1.7–6.6]). However, TAP patients showed 14% more frequent weight change compared to the control group and 3% compared to OXN (p = 0.047, large effect size = 1.007, Odd ratio of TAP vs control 1.6 [1.03–2.5] and OXN vs TAP 1.02 [0.66–1.56]).

In total, 192 commonly occurring ADRs were noted (ratio of 16 AEs: 1ADR). Mainly systems affected were 25% nervous, 17% psychiatric disorders, and 12% gastrointestinal systems without notable differences between test cases and controls (data not shown).

### *OPRM1 *and *COMT* genotypes and sex influence

The frequencies of occurrence in the study population of the *OPRM1 *(rs1799971*, A118G)* genotypes were 59% for A/A, 38% for A/G and 2% for G/G (Hardy–Weinberg equilibrium (HWE) p = 0.067). On the other hand, the frequency of the *COMT* (rs4680, *G472A*) G/G genotype was 26%, G/A 46% and A/A 28% (HWE p = 0.219).

The influence of the *OPRM1* and *COMT* genetic variants over clinical and pharmacological variables in TAP and OXN groups was analysed. In this case, no statistically significant influence was found over these variables.

**Figure 4:**
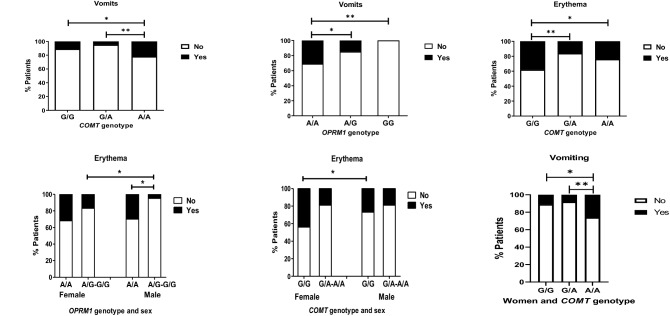
Difference of frequency of vomiting and erythema adverse events depending on *COMT* and *OPRM1* gene variants in oxycodone/naloxone case groups.

These variants’ significant impacts on frequency of occurrence of erythema (*OPRM1* A/A 31%, A/G 15% and G/G 0%, p = 0.037, medium effect size = 0.202), vomiting (*COMT* G/G 11%, G/A 5%, A/A 22%, p = 0.031, medium effect size = 0.212) and erythema (*COMT* G/G 38%, G/A 16% and A/A 24%, p = 0.031, medium effect size = 0.210) in OXN patients as can be seen in Fig. 4. Here, females reported a much higher incidence of vomiting, depending on the genotype, with *COMT* G/G reporting 11%, G/A 8%, and A/A 26% (p ≤ 0.001, medium effect size = 0.221). Incidence of erythema due a higher frequency of flushing was also found to be genotype-dependent in females (*COMT* G/G 44%, G/A 17% and A/A 26%, p = 0.025, medium effect size = 0.195). Any genetic variant analysed influence on incidence of AEs in cases.

## Discussion

Both OXN and TAP achieved a higher pain relief than other traditional opioids with a better improvement in safety profile for TAP. Here, OXN showed the highest pain relief but under a significantly higher MEDD and rates of side-effects compared to TAP. Furthermore, preliminary data indicates a lower opioid tolerability in females OXN group that could vary according to *COMT* genotype.

These results provide clear directions in terms of clinical practice. Firstly, as expected, control group reported a higher frequency of severe pain^29^ that together with the QoL, could be predictive for a higher pain relief^30^. In fact, a stronghold of our data is that provides from CNCP real-world sample of patients, that were 71% middle-aged women, under a multidrug analgesic treatment, who exhibited common pain intensity, tolerability and hospital frequentation rates^12,31^. Thus, our results would hold for similar pain patients that routinary attends PUs. Secondly, data evidenced that OXN yielded higher pain relief^32,33^ but with a higher prevalence of side-effects (including constipation rates), drug change requirements due to pain or hospital frequentations due to other causes. What’s more discuss the significant differences concerning coadjuvant medication in OXN and TAP groups, could contribute to the differences observed in analgesic tolerability that should be confirmed in further studies. Our results suggest that TAP could provide better clinical outcomes at lower costs due the lower opioid requirements and incidence of AEs^34^. It is possible that TAP’s opioid-saving effect due its mechanism of action^29^ could serve to optimize opioid rotation practices^35–37^. Here, the TAP more frequent weight change compared to other groups should be deeper analysed. Prior studies have identified an association between obesity and prescription opioid use in the US. However, the pain conditions that are factors in this association remain unestablished^38^ and this. Unfortunately, the term recorded in our study as “weight change” AE did not specify if was an increase or a loss of weight. Additional larger studies are needed to evaluate these results and, also, whether genotyping *COMT*, alone or in combination with *OPRM1,* could be potentially translated to pain practice^36^.

Our data showed a mild *COMT* genetic influence on some side-effects, such as erythema and vomiting, especially in females. Previous studies have indicated *OPRM1* and *COMT* genotypes’ significant influence on prevalence of erythema and nausea/vomiting^39^ such as for acute post-operative pain, CNCP and cancer-related pain^40^. This can be mediated by dopamine, which is an important neurotransmitter in the postrema area and vomiting centre, when it is used together with catecholamines that can modulate inflammatory processes^41^. In addition, the remarkably female predominance in this data merits further attention. Mostly of our patients were elderly women, as was previously highlighted in our pain population^42^. Literature data strongly suggest that men and women differ in their pain responses, potentially due to differences in modulation of the endogenous opioid system^43^ and sex hormones^44^, which could, in turn, have differential pharmacogenetic impacts^45,46^. Awareness about this sex influence should be emphasized in order to improve pain management.

### Limitations and strengths

Our data present some limitations. First, the lack of randomization is problematic and raises questions about bias. As noted above, important factors such as duration of pain, type of pain/diagnosis, and psychosocial factors were not controlled. This should be addressed in future studies. What’s more, the diagnoses associated with CNCP were done following clinical routines, but not with other objective measurements. This potentially clouds an understanding of what types of non-opioid analgesics as duloxetine or pregabalin could be appropriate for use. This could have introduced a bias influenced by several other variables, such as sociodemographics, that might be more relevant than pain status^47^. Second, a convenience sample was assessed based on patients attending the PU. This can affect the population representativeness, especially in genotype variables, and in this way to find significant differences. Thirdly, patients in all groups were able to take multiple opioids, and thus a host of adjuvants from various opioid combinations (such as tramadol with tapentadol) and/or from other non-analgesic prescriptions might have played a role, which was not recorded in the present study. What’s more, patients could receive other concomitant prescriptions due to their comorbidities, they might have independently contributed to the observed side-effects. Thus, AEs could not be always directly attributed to the opioid with the highest prescribed dose. This may limit conclusions related to effectiveness or side-effects. Finally, while a combination of Oxycodone with naloxone may have benefits related to gastrointestinal side effects, potential interactions between these drugs have not been contemplated in this study. All these aspects should be addressed in future studies.

## Conclusions

Taken together the findings presented here suggest that opioids of the new generation, OXN and TAP, can control pain intensity than traditional opioids used in pain treatment routines. However, OXN showed a worse tolerability and a higher health resource as compared to TAP. Additionally, *COMT* genotypes were associated with higher incidence of some opioid side-effects, especially in females. Hence, further studies are warranted to confirm and refine these results on a wider population and finally ascertain the role that pharmacogenetic in terms of improve analgesic tolerability.

## Methods

### Study design

A real-world observational and cross-sectional study was conducted from November 2014 to November 2017, using CNCP outpatients who required either OXN or TAP prescriptions. CNCP patients were recruited following their routine clinical visits for standard treatment at the Pain Unit (PU, Health Department of the Alicante-General Hospital, Spain). At the time of the enrolment, all participants received information on the design and purpose of the study and provided their written informed consent, allowing their genetic samples and electronic health records (EHRs) to be used for the research. All the methods were carried out in accordance with the ethical guidelines established in the Declaration of Helsinki. The Research Ethics Committee of the Alicante-General Hospital approved the protocol (PI2019/108, 190715), after being classify by Spanish Agency for Medicines and Health Products, which complies with the applicable STROBE guidelines.

### Participants

A total of 600 patients were pre-screened, with 7% of patients excluded (due mostly to non-chronic cancer pain or fibromyalgia). Although patients under 18 years old, pregnant women, oncologic pain or any psychiatric disorders that could interfere with the proper development of the study were excluded. Furthermore, other chronic pain syndromes of unclear pathophysiology, such as fibromyalgia, and neuropathic pain, such as painful polyneuropathy, postherpetic neuralgia, trigeminal neuralgia, and post-stroke pain, were not included^48^.

**Figure 5:**
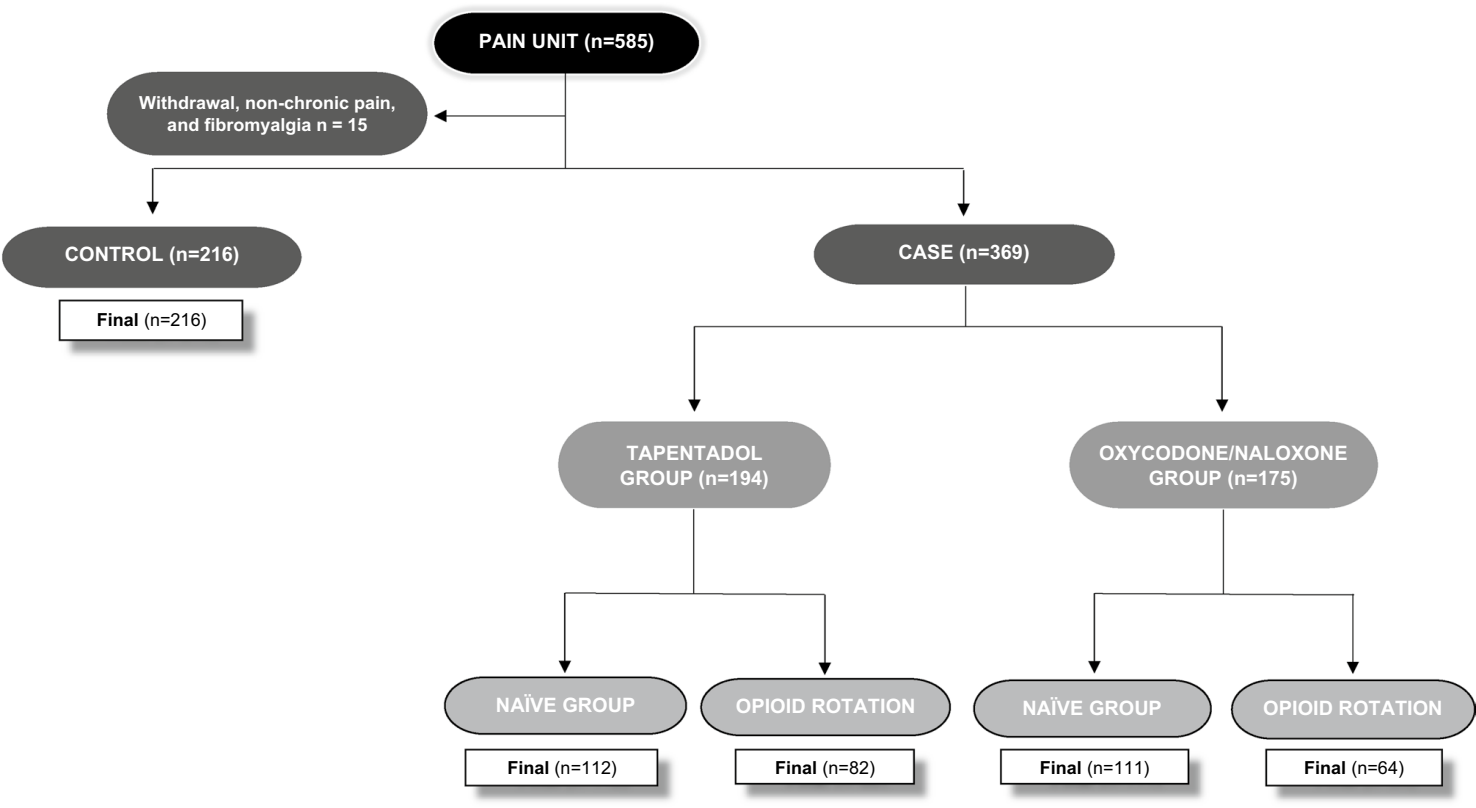
Study flow chart of patients’ selection and controls.

Finally, 585 CNCP patients (mean age 65 ± 14 years old, 71% female and all Caucasian) were included, as displayed in Fig. 5. These patients were included under the following inclusion criteria: adult men and women (≥ 18 years of age) with a stable regimen of regular opioid prescriptions (required opioid prescription for their pain) due to CNCP. There was no minimum pain score required for inclusion in the study.

Subjects were divided in two groups, cases (n = 369, under routine treatment with TAP or OXN) and controls (n = 216, other opioids except TAP or OXN). Cases could be previously naïve to opioids (n = 223) or who switched from another opioid (n = 146).

First, the researchers reviewed the schedule of PU consultations by patients weekly. Then they pre-screened patients with active OXN or TAP prescriptions and prepared the questionnaires and informed consent forms. In the case that a new patient began an OXN or TAP prescription on the day of the researchers’ visit, PU healthcare notified the researchers for their potential inclusion. For every two cases, one control was included from a concomitant observarional study^12^ (age and sex-matched patients with the same inclusion criteria yet treated with opioids different from TAP or OXN as fentanyl, morphine or buprenorphine).

The population selected as controls included a total of 1339 clinical records of 753 patients who routinely attend to PU for treat their CNCP. From this group, a subpopulation of 216 patients was extracted who were being treated with a main opioid (excluding OXN, oxycodone without combination or TAP) being main analgesic drug adjuvants prescription rate (gabapentin, duloxentin), similar to those of the test cases studied.

The lack of randomization led to the patients’ being either: 1. under OXN or TAP prescription (the test group previously naïve to opioids), 2. switched to OXN or TAP from a different opioid (minimum a month before), or 3. under another combination of opioids (e.g., morphine or fentanyl plus OXN or TAP), either due to the former’s use as a rescue medication or to aid the switching process. In any case, control group must not under treatment neither with OXN nor TAP and the opioid with the highest MEDD was designated as the main treatment.

### Procedure

A consecutive sampling method was used in ambulatory patients. When a patient met the inclusion criteria, he/she was informed about the purpose of the study by the PU healthcare staff. Then, interested individuals were attended to by the research staff for signing of the informed consent paperwork and collection of a saliva sample for the pharmacogenetic analysis.

Demographic data, pain history, drug use and medical history were recorded from EHRs. Clinical data was reported through validated scales and questionnaires completed as part of a standard clinical routine for assessing pain intensity, pain relief, QoL, and most common AEs in pain management^49^. Outcomes were assessments at a single time point where pain (intensity or relief) and QoL was asked at the present time whilst the cumulative AEs reported since last month.

Validated scales and questionnaires were used to evaluate clinical outcomes and were collected every time a patient was included at a single time point. Pain intensity, relief and QoL were measured using the visual analogue scale (VAS), consecutively by clinical routine. The VAS for each indicator consists of a 100 mm horizontal line ranging from 0 (lowest) to 100 mm (highest), where the patient points on the line to the intensity of pain or relief that he/she feels, respectively^49^. Specifically, QoL was evaluated through the VAS-EuroQol Scale, which consists of a vertical line from 0 (the worst imaginable health status) to 100 mm (the best imaginable), upon which the patient indicates his/her current health status. Likert pain intensity and relief scales were also registered (4 = extremely intense, 3 = intense, 2 = moderate, 1 = mild, 0 = none) in subsequent questionnaires.

### Safety profile

For collection of patients’ reported AEs, a questionnaire with a list of the most frequent adverse drug reactions (ADRs, selected for being “very common” or “common” on the opioids’ Summary of Product Characteristics) and a blank field to add any other AEs was collected. These AEs consisted of: sleepiness, dizziness, nausea, vomiting, constipation, itchiness, sexual dysfunction, loss of libido, weight change, headache, erythema, dry skin, dry mouth, edema, depression, insomnia, nervousness and loss of appetite. Additionally, to the questionnaire, the listed ADRs were recorded from EHRs. Clinical data of AE/ADR reporting were coded according to the medical dictionary for regulatory activities (MedDRA) and the system organ class (SOC)^50^.

In addition, the percentage of Emergency Department (ED) visits, hospitalizations, or any drug changes due to pain or other causes were registered when patients were included referred to the last month. Prescription changes included: (1) Change in any drug-dosage. (2) Product or generic brand switch. (3) Stopping medication or non-adherence, and (4) starting a new medication^51^.

The comparison between test cases’ and controls’ opioid benefit/risk profiles was defined as a balance between benefits (decrease in pain intensity and/or increase in pain relief) and tolerability in terms of number of AEs or hospital frequentation^52,53^.

### Drug prescription

Simple analgesics’ use (paracetamol, metamizole and NSAIDs) as well as prescriptions for tramadol and strong opioids like OXN, TAP or others (fentanyl, buprenorphine, morphine, or hydromorphone) were registered. In cases where different opioids were combined, oral MEDD was estimated using available references^54^.

The use of any other concomitant analgesics most widely prescribed at the PU were also registered from the institution’s EHRs: antidepressants (amitriptyline and duloxetine), anxiolytics (benzodiazepines) and gabapentinoids (pregabalin, gabapentin). For the analysis, these drugs were called “neuromodulators”, given their role as substances that alter the way nerves communicate with each other and, consequently, the overall activity level of the brain^55^.

### Genotyping

Approximately 2 ml of saliva was collected in tubes containing 6 ml of PBS. Once the saliva sample was taken, it was stored at − 80 °C until its processing. Genomic DNA was isolated using the E.N.Z.A. Forensic DNA kit (Omega bio-tek), according to the manufacturer’s instructions. Real-time polymerase chain reaction (RT-PCR) analysis was used to genotype *OPRM1 (*rs1799971, A118G) and *COMT* (rs4680, G472A) gene polymorphisms. All PCR amplifications were carried out in a RT-PCR Rotor Gene Q (Qiagen), using specific TaqMan probes MGB^®^ (Applied Biosystems). The amplification parameters were as follows: initial 10 min denaturation at 95 °C, 45 cycles for 15 s at 92 °C, 90 s at 60 °C, and 1 min final extension at 60 °C.

### Statistical analysis

Convenience sampling was considered to be more likely to represent the target population. This entailed selecting participants on the basis of availability until the final sample size was achieved^56^. The assumption of normality was validated with the Kolmogorov Smirnov test using the Lilliefors correction method. Quantitative parametric data is presented as mean ± standard deviation (SD) while non-parametric data and discrete variables are shown using their median values (interquartile range). Categorical data is expressed by percentages, among them the relative frequencies of genotypes and alleles.

Comparisons between any two given groups (case, controls) of data exhibiting parametric distributions was conducted using the independent T-test analysis, and for analyses comparing three groups an ANOVA test was carried out. Outcomes from opioid naïve vs opioid rotation patients were done to wonder any difference. Analysis of non-parametric data was done using U Mann–Whitney and Kruskal–Wallis tests for comparison between two and three groups, respectively. Comparisons for categorical data were conducted using Chi-square (χ^2^) goodness-of-fit and Fisher’s exact test. A multiple linear regression was performed to generate a predictive risk model and to analyse the influence of the following variables over pain relief: age, gender, VAS pain intensity, EQD, MEDD, number of AEs and the use of neuromodulators, anxiolytics, and analgesics.

In addition to this, the effect sizes were calculated for all the comparisons. Eta-Squared (η^2^) was used for ANOVA and Kruskal–Wallis analyses (effect size between 0.01 and 0.04 being a small effect, 0.06 and 0.11 intermediate and 0.14 and 0.2 a large effect), while for the chi-square *χ*^2^ the effect size was determined using the Cramer’s V method (effect size < 0.2 being a small, 0.2–0.6 intermediate, and > 0.6 large) and using Odd Ratios for AEs between study groups.

Observed gene frequencies were compared to expected values using the chi-square *χ*^2^ goodness-of-fit test and the Hardy–Weinberg proportion. Chi-square test analysis was conducted to compare the distribution of genotypes and alleles between the different groups. The subjects were grouped based on their genetic profiles, whether they were homozygotes or heterozygous, and whether they were carriers or non-carriers of a determinate allele. In cases of significant genetic associations, co-dominant, dominant, recessive, and over-dominant models were calculated. Sex analysis of genotypes were grouped according to the presence or absence of the mutant allele (*OPRM1* A/A vs A/G-G/G and in *COMT* gene G/G VS G/A-A/A) when frequencies of mutant alleles were low.

p-values < 0.05 were considered statistically significant. In all cases, multiple testing was adjusted using the Bonferroni correction. Analyses were carried out using the R software package version 4.0.3 and Graph Pad Prism 5.0.

## Supplementary Information

Supplementary Table S1.

Supplementary Table S2.

Supplementary Table S3.

## Data Availability

The datasets generated during and/or analysed during the current study are not publicly available due include medical information of patients but are available from the corresponding author on reasonable request.

## Abbreviations

CNCP: Chronic non-cancer pain
OXN: Oxycodone/naloxone
AEs: Adverse events
TAP: Tapentadol
OPRM1: Opioid receptor Mu
COMT: Catechol-O-methyltransferase
PU: Pain Unit
EHRs: Electronic health records
VAS: Visual analogue scale
QoL: Quality of life
ED: Emergency Department
ADRs: Adverse drug reactions
MedDRA: Medical dictionary for regulatory activities
SOC: System organ class
NSAIDs: Non-steroidal anti-inflammatory drugs
MEDD: Morphine equivalent daily dose
SD: Standard deviation
ANOVA: Analyses of variance
HWE: Hardy–Weinberg equilibrium
