# Supplementary material for: Oxycodone/naloxone versus tapentadol in real-world chronic non-cancer pain management: an observational and pharmacogenetic study
Source: Sci Rep. 2022 Jun 16;12:10126. doi: 10.1038/s41598-022-13085-5 (PMC9203709; doi:10.1038/s41598-022-13085-5)
Supplement: Supplementary file 1 — Supplementary Table S1. [file 41598_2022_13085_MOESM1_ESM.docx]

**Table 1**. Demographic and clinical data in chronic non-cancer pain patient’s tapentadol (TAP) and oxycodone/naloxone (OXN) cases groups depending on they are naïve or not to opioids.

|  | **TAP** | | **OXN** | |
| --- | --- | --- | --- | --- |
|  | **Naïve**  **(n=112)** | **ROP**  **(n=82)** | **Naïve**  **(n=111)** | **ROP**  **(n=64)** |
| **Sex (Female) (%)** | 74 | 73 | 70 | 76 |
| **Age** | 67 ± 14 | 63 ± 14 | 65 ± 13 | 61 ± 14 |
| **VAS Pain intensity**  **(0-100 mm)** | 59 ± 27 | 64 ± 24 | 64 ± 26 | 65 ± 26 |
| **Likert pain intensity (%)**  None  Mild  Moderate  Severe  Extremely Severe | 8  16  30  36  10 | 2  13  23  53  9 | 4  10  27  49  10 | 0  17  23  52  8 |
| **VAS Pain relief**  **(0-100 mm)** | 37 ± 28 | 34 ± 27 | 41 ± 30 | 39 ± 30 |
| **Likert Pain relief (%)**  None  Mild  Moderate  Severe  Extremely Severe | 21  26  36  10  7 | 26  22  39  13  0 | 18  29  36  12  5 | 27  16  45  11  2 |
| **VAS EuroQol**  **(0-100 mm)** | 45 ± 23 | 44 ± 21 | 46 ± 22 | 45 ± 24 |

Note: ROP: Rotation of opioids. Data is presented as mean ± SD or as %.
