# Supplementary material for: Oxycodone/naloxone versus tapentadol in real-world chronic non-cancer pain management: an observational and pharmacogenetic study
Source: Sci Rep. 2022 Jun 16;12:10126. doi: 10.1038/s41598-022-13085-5 (PMC9203709; doi:10.1038/s41598-022-13085-5)
Supplement: Supplementary file 2 — Supplementary Table S2. [file 41598_2022_13085_MOESM2_ESM.docx]

**Table 2.** Lineal regression of descriptive, clinical, and pharmacological parameters with pain relief as dependent variable in control, and tapentadol (TAP) and oxycodone/naloxone (OXN) cases groups.

Pharmacological parameters: Antidepressants, anticonvulsants, MEDD, number of opioids, analgesics, and anxiolytics

| **Regression Equation** | **β Coefficient** | **SE** | **T** | **Sig. T** | **95% CI for Exp (B)** | | **R^2^** | **F test** | **p** |
| --- | --- | --- | --- | --- | --- | --- | --- | --- | --- |
|  |  |  |  |  | **LL** | **UL** |  |  |  |
| **CONTROL** | | | | | | | | | |
| **Constant** | 3.152 | 0.714 | 4.414 | <0.001 | 1.74 | 4.56 | 0.221 | 9.952 | <0.001 |
| **Pain intensity** | -0.462 | 0.072 | -3.441 | <0.001 | -0.69 | -0.23 |  |  |  |
| **Age** | -0.037 | 0.009 | -3.376 | <0.001 | -0.08 | -0.008 |  |  |  |
| **TAP** | | | | | | | | | |
| **Constant** | 5.117 | 0.778 | 6.572 | <0.001 | 3.58 | 6.65 | 0.295 | 37.03 | <0.001 |
| **Pain intensity** | -0.440 | 0.076 | -5.757 | <0.001 | -0.59 | -0.29 |  |  |  |
| **Quality of life** | 0.025 | 0.009 | 2.808 | 0.005 | 0.007 | 0.04 |  |  |  |
| **OXN** | | | | | | | | | |
| **Constant** | 5.385 | 0.718 | 7.496 | <0.001 | 3.96 | 6.80 | 0.349 | 31.20 | <0.001 |
| **Pain intensity** | -0.535 | 0.073 | -7.257 | <0.001 | -0.68 | -0.39 |  |  |  |
| **Quality of life** | 0.034 | 0.008 | 3.990 | <0.001 | 0.01 | 0.05 |  |  |  |
| **Anxiolytics** | 1.309 | 0.406 | 3.217 | 0.001 | 0.50 | 2.11 |  |  |  |

**Note:** SE: Standard error; T: T-value; Sig.T: Significance of the variable; LL: Lower Limit; UL: Upper Limit; R^2^: R square adjusted; CI: Confidence Interval, MEDD: Morphine Equivalent Daily Dose
