# Supplementary material for: Oxycodone/naloxone versus tapentadol in real-world chronic non-cancer pain management: an observational and pharmacogenetic study
Source: Sci Rep. 2022 Jun 16;12:10126. doi: 10.1038/s41598-022-13085-5 (PMC9203709; doi:10.1038/s41598-022-13085-5)
Supplement: Supplementary file 3 — Supplementary Table S3. [file 41598_2022_13085_MOESM3_ESM.docx]

**Table 3.** Adverse event patients self-reported and Adverse Drug Reactions suspected in in control, and tapentadol (TAP) and oxycodone/naloxone (OXN) cases groups.

| **Adverse events (%)** | **CONTROL (n=216)** | **CASE** | | **p-value**  **Effect size** | **Odd ratio (confidence interval)** |
| --- | --- | --- | --- | --- | --- |
|  |  | **TAP**  **(n=194)** | **OXN**  **(n=175)** |  |  |
| Xerostomia | 63 | 64 | 68 | 0.601  0.412 ^I^ | 1.00 (0.65-1.54)^1^  1.18 (0.76-1.84)^2^  1.24 (0.8-1.92)^3^ |
| Constipation | 55 | 51 | **68^†^*** | <0.001  **2.4** ^I^ | 1.07 (0.71-1.62)^1^  **2.4 (1.6-3.8)**^2^  **2.3 (1.49-3.53)^3^** |
| Nervousness | **55**** | 42 | 35 | 0.002  **1.461** ^I^ | 0.60 (0.39-0.91)^1^  0.55 (0.33-0.87)^2^  **1.32 (1.02-1.70)^3^** |
| Dry skin | 36 | 38 | 44 | 0.070  **0.94** ^I^ | 1.30 (0.84-2.01)^1^  1.68 (1.09-2.62)^2^  1.29 (0.84-1.97)^3^ |
| Depression | 40 | 34 | 39 | 0.281  **0.65** ^I^ | 0.69 (0.45-1.05)^1^  0.83 (0.54-1.27)^2^  1.20 (0.78-1.85)^3^ |
| Insomnia | 26 | 36 | 35 | 0.068  0.179 ^I^ | 1.03 (0.67-1.59)^1^  1.06 (0.68-1.64)^2^  1.02 (0.66-1.57)^3^ |
| Weight change | 25 | **39*** | 36 | 0.047  **1.007** ^I^ | **1.60 (1.03-2.50)^1^**  1.64 (1.05-2.56)^2^  1.02 (0.66-1.56)^3^ |
| Somnolence | 49 | 41 | 48 | 0.514  **0.588** ^I^ | 0.99 (0.65-1.50)^1^  1.34 (0.88-2.04)^2^  1.35 (0.89-2.06)^3^ |
| Loss of appetite | 25 | 30 | 35 | 0.357  **0.586** ^I^ | 1.09 (0.69-1.71)^1^  1.09 (0.68-1.73)^2^  0.99 (0.63-1.75)^3^ |
| Dizziness | 40 | 34 | 36 | 0.377  **0.57** ^I^ | 0.78 (0.51-1.20)^1^  0.87 (0.57-1.34)^2^  1.11 (0.72-1.72)^3^ |
| Headache | 26 | 33 | 36 | 0.250  **0.68** ^I^ | 1.11 (0.71-1.73)^1^  1.43 (0.92-2.23)^2^  1.29 (0.83-1.99)^3^ |
| Loss of libido | 17 | 24 | 24 | 0.478  **0.495 ^I^** | 1.32 (0.80-2.18)^1^  1.34 (0.80-2.22)^2^  1.10 (0.62-1.64)^3^ |
| Pruritus | 27 | 25 | 26 | 0.610  0.406 ^I^ | 0.78 (0.49-1.25)^1^  0.84 (0.53-1.35)^2^  1.07 (0.66-1.73)^3^ |
| Nausea | 25 | 20 | 24 | 0.645  0.382 ^I^ | 0.96 (0.57-1.59)^1^  1.59 (0.98-2.50)^2^  1.65 (1.01-2.70)^3^ |
| Edema | 7 | 18* | **21*** | 0.001  **1.498** ^I^ | 2.65 (1.34-5.25)^1^  **3.40 (1.74-6.66)^2^**  1.28 (0.75-2.17)^3^ |
| Erectile dysfunction | 15 | 13 | 10 | 0.480  **0.494 ^I^** | 0.92 (0.50-1.71)^1^  0.69 (0.36-1.34)^2^  0.74 (0.38-1.45)^3^ |
| Erythema | 12 | 11 | **24^†^*** | 0.001  **1.46** ^I^ | 0.96 (0.50-1.83)^1^  **2.37 (1.34-4.21)^2^**  **2.47 (1.39-4.37)^3^** |
| Vomiting | 10 | 7 | 11 | 0.407  **0.547** ^I^ | 0.77 (0.36-1.65)^1^  1.24 (0.61-2.50)^2^  1.61 (0.77-3.37)^3^ |
| **Total** | **4 [0-6]**** | **5 [2-8]** | **6 [3-9]** ^†^** | <0.001  0.07 ^II^ | NA |

**Note:** Data is presented as median of AEs per patient and %.

Comparison cases vs. control, p<0.05 (*). P< 0.001 (**) and (†) p<0.05 tapentadol vs. oxycodone/naloxone, cell in grey. Chi-square χ2 the effect size was determined using I Cramer’s V (effect size<0.2 small, 0.2<effect size<0.6 intermediate and 0.6<effect size large effect). II Eta squared for Kruskal Wallis Test (effect size of 0.01-0.04 small, 0.06-0.11 intermediate and 0.14-0.2 large effect). Large effect size is written in bold font. Odd Ratio: 1: Control vs TAP. 2: Control vs OXN. 3: TAP vs OXN)

NA: not applicable
